# Data on eNOS T786 and G894T polymorphisms and peripheral blood eNOS mRNA levels in Sickle Cell Disease

**DOI:** 10.1016/j.dib.2016.11.082

**Published:** 2016-11-28

**Authors:** Iakovos Armenis, Vassiliki Kalotychou, Revekka Tzanetea, Panagoula Kollia, Zoi Kontogeorgiou, Dimitra Anastasopoulou, Marina Mantzourani, Michael Samarkos, Konstantinos Pantos, Kostas Konstantopoulos, Ioannis Rombos

**Affiliations:** a 1st Department of Internal Medicine, “Laiko” Hospital, NKUA, Medical School, Athens, Greece; b Metropolitan Hospital, Athens, Greece; c Department of Biology, NKUA, Athens, Greece; d Department of Pharmacology, NKUA, Medical School, Athens, Greece; e Department of Hematology, “Laiko” General Hospital, NKUA, Medical School, Athens, Greece

## Abstract

In this article, we present data on endothelial Nitric Oxide Synthase (*eNOS*) gene T786C and G894T polymorphisms in Greek steady-state Sickle Cell Disease patients in comparison to healthy controls. Moreover, *eNOS* mRNA levels were determined in peripheral blood samples from 18 patients and 9 controls. This article complements our recently published article named “Prognostic value of *eNOS* T786C and G894T polymorphisms in Sickle Cell Disease” (I. Armenis, V. Kalotychou, R. Tzanetea, Z. Kontogeorgiou, D. Anastasopoulou, M. Mantzourani, M. Samarkos, K. Pantos, K. Konstantopoulos, I. Rombos, 2016) [1].

**Specifications Table**

**Table:** 

Subject area	*Genetics, Hematology*
More specific subject area	*eNOS T786C, G894T polymorphisms, eNOS mRNA levels*
Type of data	*Table, image, figure*
How data was acquired	*Venous blood from 79 patients and 48 controls*
Data format	*Analyzed*
Experimental factors	*Venous blood was collected from steady-state Sickle Cell Disease patients and healthy controls*
Experimental features	*DNA extraction, RNA extraction, PCR, RFLPs, Sanger sequencing, qRT-PCR*
Data source location	*Greek non-consanguineous Sickle Cell Disease patients*
Data accessibility	*Data is with this article*

**Value of the data**

• Genotype and allele distribution of *eNOS* T786C and G894T polymorphisms were studied for the first time in Greek Sickle Cell Disease patients.
• This is the first study to find higher prevalence of the C allele in comparison to T for the -786 position in Sickle Cell Disease.
• Peripheral blood *eNOS* mRNA levels, as an index of *eNOS* expression, were studied and analyzed according to the presence or absence of Sickle Cell Disease and *eNOS* promoter T786C polymorphism.

## 1 Data

**Fig. 1:**
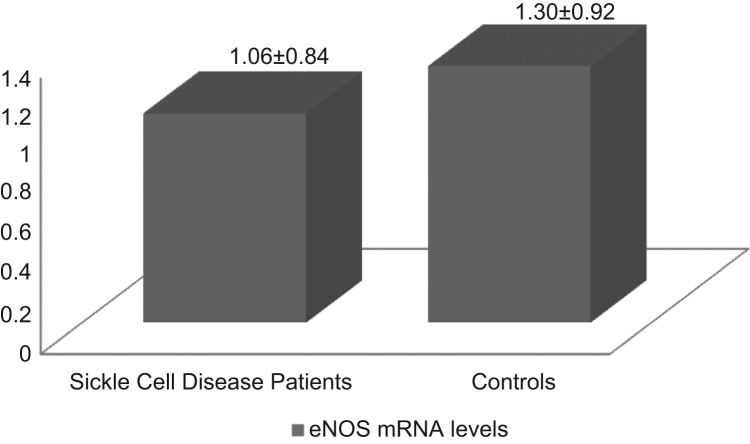
*eNOS* mRNA levels in Sickle Cell Disease patients (*n*=18) and healthy controls (*n*=9), relative to *GAPDH* mRNA. Sickle Cell Disease patients: 1.06±0.84, Controls: 1.30±0.92, *P*=0.105.

**Fig. 2:**
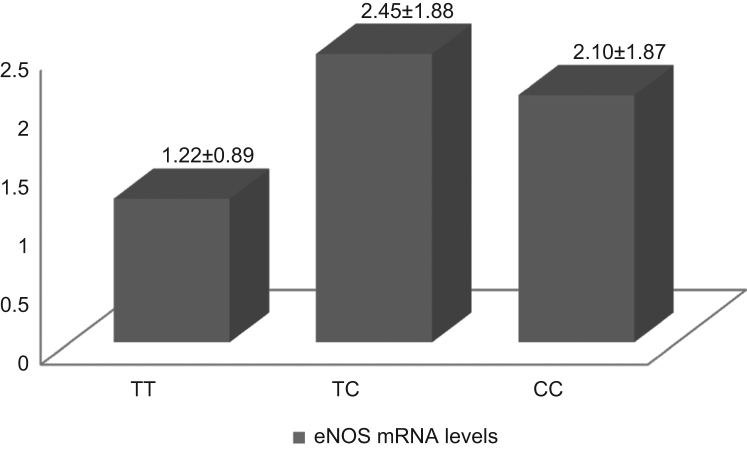
*eNOS* mRNA levels relative to GAPDH in Sickle Cell Disease patients, according to their *eNOS* T786C genotype: TT: 1.22±0.89, TC: 2.45±1.88, CC: 2.10±1.87, *P*=0.454.

Genotype and allele distribution for polymorphisms T786C and G894T in patients and controls are presented in Table 1, Table 2 respectively. No statistically significant differences were obtained [1]. *eNOS* mRNA levels normalized to *GAPDH* are presented in Fig. 1 (Sickle Cell Disease patients relative to controls) and Fig. 2 (patients of *eNOS* 786TC and CC genotypes relative to patients of TT genotype).

## 2 Experimental design, materials and methods

### 2.1 Study population

Seventy nine consecutive Greek SCD patients (mean age: 48.8±11.5 years, 25–76 years, 25 male, 54 female) and forty eight Greek healthy controls were enrolled. The study was approved by the Hospital Ethics Committee in accordance to Helsinki Declaration. All patients were at steady state at the time of sample collection (no painful crises or other acute complications during the last three months) and they had not been transfused for the last three months.

### 2.2 DNA extraction and *eNOS* genotyping

Genomic DNA was isolated from peripheral blood using the PureLink Genomic DNA extraction kit (Invitrogen). Nucleotide substitutions T786C and G894T were detected using Sanger sequencing and PCR-RFLPs techniques respectively. The PCR primers used were as follows:

for T786C: 5′*ATGCTCCCACCAGGGCATCA*3′ (forward),

5′*GTCCTTGAGTCTGACATTAGGG*3′ (reverse) covering a fragment of 237 bp.

and for G894T: 5′*CATGAGGCTCAGCCCCAGAAC*3′ (forward), 5′*AGTCAATCCCTTTGGTGCTCAC*3′ (reverse) covering a fragment of 206 bp.

**Image 1:**
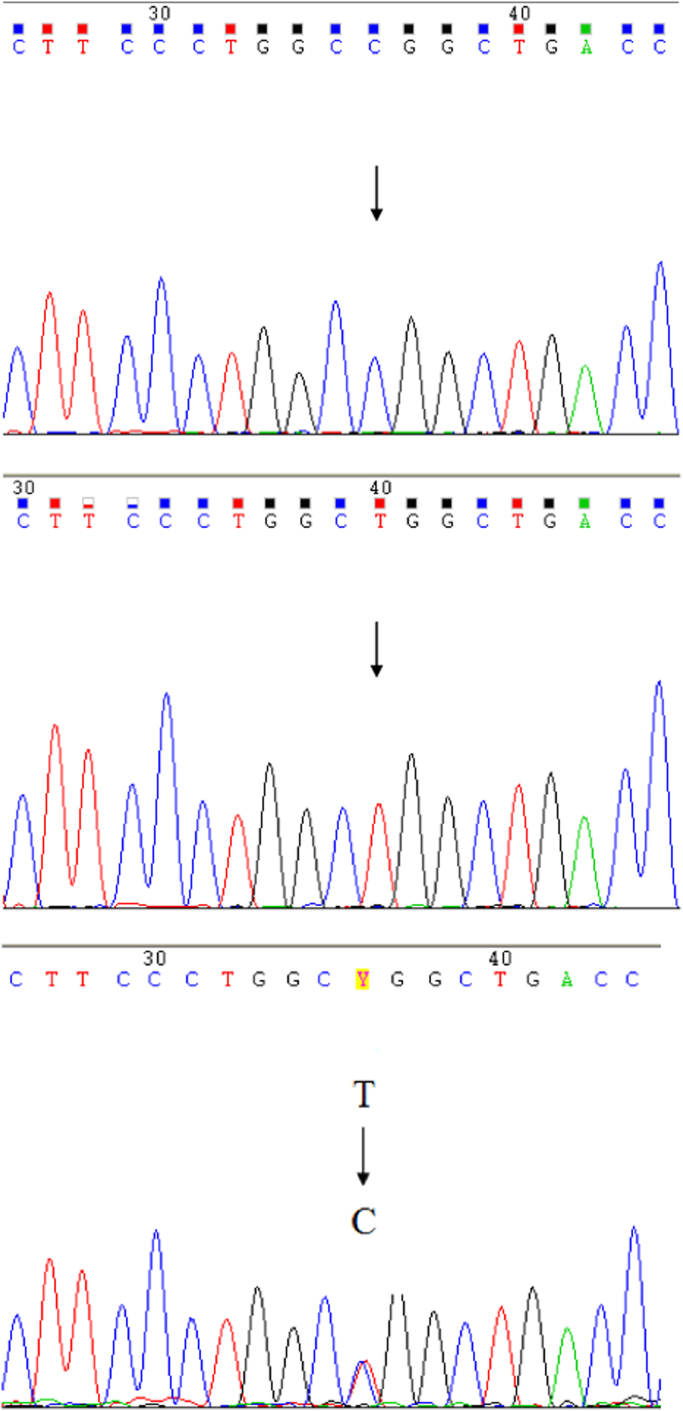
Sanger sequencing results from 3 samples of 786CC, TT and TC genotype respectively.

**Image 2:**
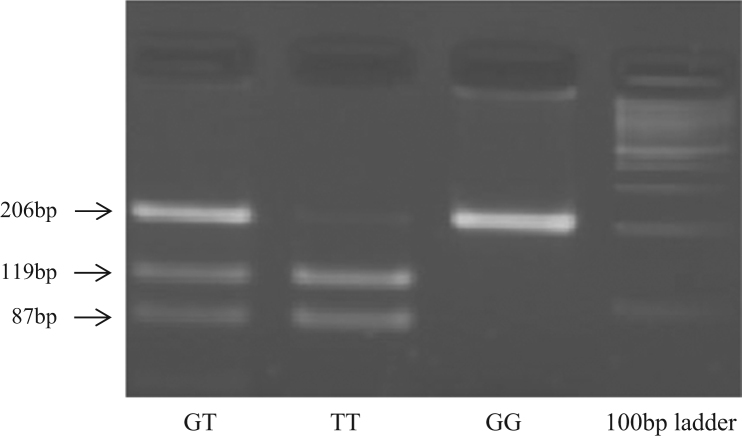
Agarose gel electrophoresis. Three samples were digested with *MboI* restriction enzyme, after PCR amplification for *eNOS* G894T polymorphism.

PCR conditions for T786C were: 92 °C for 15 s, 62 °C for 30 s, and 72 °C for 30 s repeated for 34 cycles. For G894T, we applied the previous conditions decreasing the annealing temperature to 58 °C. Presence of 786C polymorphism was determined by Sanger sequencing of the PCR product using the above mentioned PCR primers, as shown in Image 1. The 894T polymorphism was detected by PCR-RFLPs, digesting with the appropriate restriction enzyme, MboI, revealing two fragments of 119 base pairs and 87 base pairs, as shown in Image 2.

### 2.3 RNA extraction and quantitative real-time PCR

Eighteen SCD patients (5 male and 13 female) underwent eNOS expression study. Total RNA was isolated from peripheral blood of the above patients and 9 controls (4 male and 5 female), none of them smoking or receiving drugs apart from folate supplementation and acetylsalicylic acid. The RNeasy Mini Kit and Qiashredder homogenizer (Qiagen) after selective lysis of erythrocytes were applied. One microgram of total RNA was reverse-transcribed using the iScript™ cDNA SynthesisKit (Bio-Rad). Fifty nanograms of cDNA were used for eNOS quantification on a CFX-96 real-time PCR (Bio-Rad CFX96TouchTM) using hydrolysis probes for *eNOS* gene and for *GAPDH* as a reference gene (Applied Biosystems). Amplification was performed with the appropriate cycling parameters, 95 °C for 10 min followed by 40 cycles of 95 °C for 15 s and 60 °C for 1 min. Measurements were performed in duplicate using the iTaq™ Universal Probes supermix (Biorad). Relative gene expression data were analyzed using the Livak 2^−ΔΔCt^ method.

### 2.4 Statistical analysis

Frequencies of genotypes and clinical characteristics were compared using Pearson׳s chi-square test and Fischer-exact test, where applicable. The general, dominant and recessive genetic models, as well as allele frequencies were used to compare patients and normal controls. Quantitative data were expressed as mean±S.D. and were compared with Analysis of Variance (ANOVA) or Mann–Whitney U test, whether applicable. Levene׳s *F* test was applied to estimate equality of variances and Kolmogorov–Smirnof test to assess deviation from normal distribution. A *P*-value lower than 0.05 was considered as statistically significant.

## Figures and Tables

**Table 1:** Genotype and allele frequencies for *eNOS* T786C polymorphism in Sickle Cell Disease patients and controls.

	Frequencies		*P*
	Sickle Cell Disease patients (*n*=79)	Controls (*n*=48)	
Genotypes			
TT vs TC+CC	21.5% vs 78.5%	10.4% vs 89.6%	0.173
CC vs TT+TC	46.8% vs 53.2%	47.9% vs 52.1%	0.906
			
Alleles			
T vs C	37.3% vs 62.7%	31.3% vs 68.7	0.324

**Table 2:** Genotype and allele frequencies for *eNOS* G894T polymorphism in Sickle Cell Disease patients and controls.

	Frequencies		*P*
	Sickle Cell Disease patients (*n*=79)	Controls (*n*=48)	
Genotypes			
GG vs GT+TT	50.6% vs 49.4%	39.6% vs 60.4%	0.226
TT vs GG+GT	19.0% vs 81.0%	12.5% vs 87.5%	0.340
			
Alleles			
G vs T	65.8% vs 34.2%	63.6% vs 36.4%	0.712
